# Molecular interactions between the soilborne pathogenic fungus *Macrophomina phaseolina* and its host plants

**DOI:** 10.3389/fpls.2023.1264569

**Published:** 2023-09-14

**Authors:** Miwa Shirai, Thomas Eulgem

**Affiliations:** Center for Plant Cell Biology, Institute for Integrative Genome Biology, Department of Botany & Plant Sciences, University of California at Riverside, Riverside, CA, United States

**Keywords:** *Macrophomina phaseolina*, pathogenicity, crop destroyer, crop diseases, plant immunity, host defense, root-specific defense, plant defense signaling

## Abstract

Mentioned for the first time in an article 1971, the occurrence of the term “*Macrophomina phaseolina*” has experienced a steep increase in the scientific literature over the past 15 years. Concurrently, incidences of *M. phaseolina*-caused crop diseases have been getting more frequent. The high levels of diversity and plasticity observed for *M. phasolina* genomes along with a rich equipment of plant cell wall degrading enzymes, secondary metabolites and putative virulence effectors as well as the unusual longevity of microsclerotia, their asexual reproduction structures, make this pathogen very difficult to control and crop protection against it very challenging. During the past years several studies have emerged reporting on host defense measures against *M. phaseolina*, as well as mechanisms of pathogenicity employed by this fungal pathogen. While most of these studies have been performed in crop systems, such as soybean or sesame, recently interactions of *M. phaseolina* with the model plant Arabidopsis thaliana have been described. Collectively, results from various studies are hinting at a complex infection cycle of *M. phaseolina*, which exhibits an early biotrophic phase and switches to necrotrophy at later time points during the infection process. Consequently, responses of the hosts are complex and seem coordinated by multiple defense-associated phytohormones. However, at this point no robust and strong host defense mechanism against *M. phaseolina* has been described.

## Introduction

*Macrophomina phaseolina* (Tassi) Goid is a soilborne fungal plant pathogen of the ascomycetes family Botryosphaeriaceae that causes charcoal rot, a root and stem disease characterized by black specs in the host tissue. It is distributed across the globe and is particularly problematic in arid climates (Kaur et al., 2012a). Charcoal rot symptoms are known to be more severe under heat and drought stress (Mughogho and Pande, 1983; Fang et al., 2011). More than 500 plant species have been reported to be susceptible to this pathogen, including many economically important crops such as canola, maize, sesame and soybean (Su et al., 2001; Gaetán et al., 2006; Saleh et al., 2010). Moreover, *M. phaseolina* is capable of human infection in immunosuppressed individuals, which highlights the range of host environments where it can thrive (Tan et al., 2008; Arora et al., 2012). Pathogens like *M. phaseolina* with a wide host range are described as “generalists”. *M. phaseolina* is difficult to eliminate from infected fields due to its production of microsclerotia, which are dark-colored asexual propagation structures that can survive for many years in soil without a host. Originally *M. phaseolina* was considered to be a necrotrophic pathogen, but detailed analysis of the early stages of infection in soybean revealed the presence of a distinct biotrophic phase of up to 36 hours preceding the onset of necrotic plant tissue collapse (Chowdhury et al., 2017). *M. phaseolina* is therefore often characterized as a hemibiotroph along the likes of *Phytophthora infestans* and *Fusarium oxysporum.* The asymptomatic biotrophic infection phase makes early identification of disease in the field difficult. Several factors seem to contribute to a growing number of *M. phaseolina*-related crop disease incidents. For example, strawberry collapse due to charcoal rot in the U.S. began in 2005, and by 2014 this disease was confirmed in most strawberry-growing counties (Koike et al., 2016). This rise of strawberry field infestation is correlated with high temperatures and drought, as well as the ban on methylbromide fumigation (Muchero et al., 2011). Along with its increased impact on crop production studies on mechanisms of *M. phaseolina* pathogenicity and host immunity against this root pathogen are emerging. However, our understanding of these processes is still fragmentary.

## *M. phaseolina*: a global “crop destroyer”

### Agricultural impact

Due to its wide host range, ubiquitous global distribution, and increasing impact on agriculture, *M. phaseolina* has been dubbed a “global crop destroyer”. Disease caused by *M. phaseolina* is most often referred to as charcoal rot, but this pathogen is also known to cause wilting, stem canker, seedling blight, rot, and damping-off (Singh et al., 1990; Kaur et al., 2012b). These contribute to yield loss, reduction in seed quality, and viability of infected plants (Mughogho and Pande, 1983). Moreover, the severity of symptoms is correlated with high temperatures and low soil water potential conditions. Thus, plants growing in (sub)-tropical arid regions are at higher risk of succumbing to disease caused by this pathogen (Mayek-Pérez et al., 2002; Chamorro et al., 2015; Lodha and Mawar, 2020). Pandey and Basandrai reviewed the current literature on factors related to climate change that could potentially worsen *M. phaseolina* infestation (Pandey and Basandrai, 2021). Both the visible traits and genetic architecture of this pathogen, which are further described below, show that *M. phaseolina* as a species is well adapted to diverse climates and equipped to withstand environmental change.

Many important crops including grains and legumes are included among the wide host range of this pathogen (Pandey and Basandrai, 2021). Known hosts of *M. phaseolina* such as alfalfa, maize, canola, soybean, and sunflower have vast global economic implications, while others like cassava, chickpea, and mungbean are critical for regional food security (Ammon et al., 1974; Bashir and Malik, 1988; Kaiser and Das, 1988; Msikita et al., 1998; Pratt et al., 1998; Gaetán et al., 2006; Weems et al., 2011; Lakhran et al., 2018). The combined estimated loss of soybean yields in ten countries due to charcoal rot exceeded 2 million metric tons during 1998, ranking third among the most common soybean diseases (Wrather et al., 2001). Furthermore, charcoal rot ranked second for economic losses per hectare in the U.S. across two decades and combined with soybean cyst nematode accounted for 33% of the total economic losses (Bandara et al., 2020).

*M. phaseolina* also poses a great threat to specialty crops, such as strawberry, pistachio, and sunflower (Mertely et al., 2005; Bokor, 2007; de los Santos et al., 2019; Nouri et al., 2020). Research on specialty crops is often underfunded and disproportionately lacking in comparison to the steady rise in value of production, despite the inherently high risk involved in marketing fresh produce (Alston and Pardey, 2008; Neill and Morgan, 2021). For example, strawberries ranked fourth among the top California crops produced in 2017, with a value of $3.10 billion (California Department of Food and Agriculture; Koike et al., 2016). Strawberries are highly susceptible to *M. phaseolina*. Fumigation with methylbromide and chloropicrin served as a common method of pathogen control. In 2005, the agricultural use of methylbromide was phased out. Since then, the relationship between yield and area of strawberry production reversed, as yields stagnated and new incidences of charcoal rot increased (Holmes et al., 2020).

### Disease cycle

*M. phaseolina* is most often characterized by its production of microsclerotia, or small sclerotial bodies 200–600 μm in diameter, which appear as dark specs in infected plant tissue (Jackson and Jaronski, 2009). Microsclerotia are spherical clusters of hyphal cells, each with the ability to germinate and propagate when appropriate environmental conditions are met. They can remain dormant in dead host tissue or soil for an extended period of time, becoming the primary inoculum for the next growing season (Short et al., 1980). On potato dextrose agar (PDA), microsclerotium formation can be identified in hosts as early as 24–30 hours post-inoculation (Wyllie and Brown, 1970). Pycnidia, which are asexual reproductive structures that form spores, have also been documented for this fungus, but are rarely observed *in vitro* (Ma et al., 2010).

**Figure 1:**
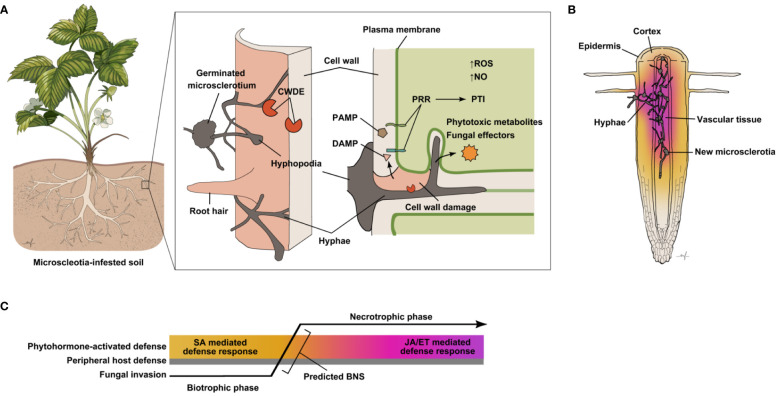
Presumed events and molecular interactions determining the outcome of host infection by *M. phaseolina*. **(A)** Schematic diagram of presumed *M. phaseolina* pathogenesis process and host defense responses. Microcsclerotia (asexual propagation structures) in the soil germinate under suitable conditions emitting hyphae that extend until a host plant, typically its root, is detected. The first line of defense a host plant possesses is its cell wall, which mainly serves as a passive physical barrier. *M. phaseolina* hyphopodia have been observed at sites of hyphal penetration of the host epidermis (Ammon et al., 1974; Bressano et al., 2010; Chowdhury et al., 2014). The *M. phaseolina* genome encodes a large number of cell wall degrading enzymes (CWDEs), predicted to aid in breaching host cell walls. Plant cell wall damage leads to the release of cell wall-derived molecules, some of which may be detected by host pattern recognition receptors (PRRs) as damage-associated molecular patterns (DAMPs) triggering pattern-triggered immunity (PTI). Host PRRs may also trigger PTI upon recognition of pathogen-associated molecular patterns (PAMPs), molecules directly derived from the pathogen. In the initial stages of infection, hyphae generally stay intercellular. At later stages hyphae can also be seen invaginating host cells, while remaining enveloped in host plasma membranes (Chowdhury et al., 2017). The *M. phaseolina* genome also encodes predicted effector proteins and has been shown to produce several phytotoxic compounds, which may further disrupt host cell function and defense processes. Whether such molecules are secreted into the host tissue to aid pathogenicity remains to be confirmed. Increased accumulation of reactive oxygen species (ROS), as well as nitric oxide (NO) have been observed in many plant hosts infected with *M. phaseolina*. While both ROS and NO accumulation are known defense responses in plants, it has also been suggested that *M. phaseolina* is capable of NO production (Sarkar et al., 2014). **(B)** *M. phaseolina* continues to invade host root tissue, spreading profusely through various tissue types and forming new microsclerotia. Common causes of plant collapse for *M. phaseolina*-infected plants are blockage and necrosis of vascular tissue. Activation of host defense by *M. phaseolina* described in the literature thus far represents that of whole roots, which includes different cell types at various stages of infection. The color gradient represents that photohormone-mediated defense responses are likely to vary and differ between cell types and infection stages. **(C)** Predicted sequence of events during *M. phaseolin*a invasion of host plant tissue, spatially and temporally depicted. Defense activation in the host and fungal transition from biotrophy to necrotrophy, as it has been observed through current methods of detection, is averaged across space from cell to cell and time relative to the infection progress. Hence, phytohormone-mediated defense response is depicted as a gradient. As *M. phaseolina* penetrates peripheral host tissues it is presumably in its biotrophic phase eliciting SA-mediated host immune responses. A biotrophic to necrotrophic switch (BNS) likely occurs after hyphae further progress into host tissues leading to activation of JA and ET-mediated defense signaling.

Microsclerotia germinate at a temperature range of 28–35°C, as the emerging mycelium extends radially until it identifies host tissue (Ammon et al., 1974; Mihail and Taylor, 1995). Appressoria-like structures, or hyphopodia, form when hyphal tips recognize host root cells, often penetrating the plant tissue between epidermal cells, before colonization is established in the middle lamella and intercellular space (Ammon et al., 1974). Early stages of infection remain mostly intercellular, but during host cell penetration the hyphae can invaginate the host plasma membrane creating an interface similar to that of haustoria of biotrophic pathogens (**Figure 1A**). Moreover, formation of intracellular vesicles, which is a characteristic of early hemibiotrophic infection, has been observed in the cortical tissue of infected soybean (Chowdhury et al., 2017). Production of pectolytic and cellulolytic enzymes by the pathogen has been observed both *in vivo* and *in vitro*, but it is not certain during which phases of the infection process this occurs (Radha, 1953; Chan and Sackston, 1969; Chan and Sackston, 1970). Development of thin, filamentous hyphae is thought to be an indicator of a biotrophy to necrotrophy switch (BNS), as subsequently, tissue begins to necrose (Chowdhury et al., 2017). *M. phaseolina* mycelia eventually colonize the host’s vascular tissue, and the formation of microsclerotia in the xylem vessels leads to blockage and consequently wilting of the host (Abawi and Corrales, 1990; Mayek-Pérez et al., 2002; **Figure 1B**). A deeper understanding of the initial stages of *M. phaseolina* infection may provide us with clues as to which modes of plant defense are effective against this pathogen.

### Strategies for control

*M. phaseolina* is adapted to survive in diverse environmental conditions, including sodium salt concentrations of 1 to 8%, and pH ranges of 3.5 to 10 (Islam et al., 2012). Its microsclerotia can persist in soil for up to 15 years. There is no universal, economically, and environmentally reasonable protocol for eradicating this pathogen from an infected field. What is known is that there is a clear relationship between inoculum concentration, disease occurrence, and yield loss. Numerous strategies to suppress charcoal rot have been tested, and the results have been thoroughly reviewed by authors such as Lodha and Mawar (2020) and Marquez et al. (2021). Current approaches to controlling this disease can be grouped into four categories: (1) Agronomic practices, (2) Chemical control by using fungicides or herbicides, (3) Host genetic resistance, and (4) Biological control (Marquez et al., 2021).

While certain combinations of agronomic practices showed some promise, none of these measures proved to be fully sufficient. The situation seems to be similar for fungicides against charcoal rot. Although highly effective in controlling *M. phaseolina*, methylbromide has been phased out throughout the world per the “Montreal Protocol Guidelines on Substances that Deplete the Ozone Layer”, released in 1991 (Chamorro et al., 2016; Guthman, 2017). Fumigation of strawberry fields with other chemicals continues in California, and in fact, chloropicrin use has increased significantly in correlation to the drop in methylbromide use, as it is less effective alone (Guthman, 2017). Although alternatives to methylbromide, including chloropicrin, have been shown to reduce *M. phaseolina* density and plant mortality in the field, crop production cost and chemical use have only increased (Chamorro et al., 2016; Guthman, 2017).

Unlike resistance against many foliar pathogens, host resistance against *M. phaseolina* seems not to be mediated by individual disease resistance (R)-genes, and multiple genetic loci seem to make combined quantitative contributions in protecting plants against this root pathogen (Marquez et al., 2021). Only gradual differences in quantitative resistance against *M. phaseolina* have been observed among different varieties of certain crop species, such as cowpea, soybean, or strawberry (Muchero et al., 2011; Reznikov et al., 2019; Gomez et al., 2020). While the underlying quantitative trait loci can be useful for breeding, genetic engineering can also result in crop varieties with enhanced protection against *M. phaseolina*. For example, transgenic jute lines expressing the *rice chitinase* (*chi11*) gene showed significantly smaller lesion sizes and improved fiber yield in comparison to the wild type when infected with *M. phaseolina* (Majumder et al., 2018). However, efforts to combat *M. phaseolina* by transgenic crop lines have been scarce. This approach has been used more so to identify genes and pathways important for host resistance (Lygin et al., 2013; Dabi et al., 2020).

Biological control strategies have shown some promise in protecting crops against *M. phaseolina*. Dhingra et al. (1976) found that increasing organic matter in soil reduces *M. phaseolina* colonization of soybean and maize stems, likely due to the increased presence of other microbes. The implication that *M. phaseolina* has low competitive saprophytic ability has been tested and confirmed with multiple fungal and bacterial biocontrol agents (BCAs). For example, several species of *Bacillus* have been reported to antagonize *M. phaseolina* growth both *in vitro* and *in vivo* and to suppress disease symptoms in soybean, common bean and cowpea (Yasmin et al., 2020; Bojórquez-Armenta et al., 2021; Rangel-Montoya et al., 2022). Other rhizospheric bacteria such as fluorescent *Pseudomonas* spp. have also been identified as *M. phaseolina* BCAs (Gupta et al., 2002; Das et al., 2008). Many of these rhizobacterial species also have plant growth-promoting properties.

The genus *Trichoderma* is a well-known fungal BCA (Lorito et al., 2010). Indeed, examples like *T. viride* and *T. harzianum* successfully inhibit the growth of *M. phaseolina* and further have positive effects on plant growth (Sankar and Sharma, 2001; Khaledi and Taheri, 2016; Khalili et al., 2016). Interestingly, it has been shown that the combined application of *T. harzianum* and arbuscular mycorrhizal fungi (AMF) allows for AMF colonization in non-host *Brassica* plants, including *Arabidopsis thaliana*, and increases silique numbers (Poveda et al., 2019). Additionally, AMF co-treatment with *M. phaseolina* resulted in a much lower number of up-regulated genes in comparison to the application of the pathogen alone. Pre-mycorrhized plants also exhibited greater expression of serine carboxipeptidase-like (SCPL) and lectin genes, which have been suggested to prime the plant for pathogen attack (Marquez et al., 2018; Marquez et al., 2021).

Despite the growing interest in reducing agrochemical inputs, the biopesticide market constitutes only a small fraction (6.4% in 2018) of the total pesticide market (Pal and McSpadden Gardener, 2006; Regnault-Roger, 2020). Obstacles that remain to be addressed, such as storage, lack of commercialized products, narrow target application, and limited user awareness have hindered widespread adoption of biological control strategies.

## *M. phaseolina*’s toolkit for pathogenicity

### Pathogen variability

Comparisons between *M. phaseolina* isolates across host species and geographical distances have revealed high levels of morphological, physiological, pathogenic, and genetic variation. Despite this, the genus *Macrophomina* is monospecific, meaning that it constitutes a single species. Classification of *M. phaseolina* isolates based on chlorate sensitivity was previously proposed by Pearson et al. (1986). Chlorate is an analogue of nitrate and can be toxic to plants and fungi when reduced to chlorite via the nitrate reductase pathway. Since *M. phaseolina* isolates from corn were chlorate resistant and those from soybean were chlorate sensitive, it was suggested that the presence or absence of the nitrate reductase pathway could be a marker for host-specific strains (Pearson et al., 1986; Pearson et al., 1987). However, subsequent reports using this approach have had mixed results. Mihail and Taylor (1995) were unable to confirm relationships between the chlorate phenotype and origin host species when comparing 114 isolates. Su et al. (2001) found that both host species and location (root or soil) of a given isolate had effects on chlorate sensitivity, albeit at varying levels.

Random amplified polymorphic DNA (RAPD) analysis can also be used to genetically differentiate *M. phaseolina* isolates by host species (Su et al., 2001). Jana et al. (2005) also successfully used two different kinds of markers derived from repetitive sequences to fingerprint and differentiate isolates from soybean, cotton, and chickpea. Baird et al. (2010) surveyed 109 isolates across the U.S. using 12 simple sequence repeats and found them to group into clusters based on both the location and host from which these isolates were found, but exceptions were also not uncommon. There is no evidence of sexual reproduction in *M. phaseolina* (Marquez et al., 2021). In a study that examined isolates across host plant families and geographical distances, hyphal fusions were observed frequently enough (64.3%) for the authors to conclude that there are no genetic barriers to ​​nonsexual genetic exchange amongst *M. phaseolina* isolates (Mihail and Taylor, 1995). The potential for gene flow across populations and the movement of agricultural products including soil likely contribute to the variability seen in this species. As nearly 4% of the annotated *M. phaseolina* genome encodes transposable elements, genetic transposition is another potential avenue for diversity due to the introduction of novel mutations, gene duplications, and horizontal gene transfers (Islam et al., 2012). Understanding *M. phaseolina* variance is important for developing resistant crop genotypes and highlights the importance of experimental design in studying this pathogen.

### Host specialization

There are many conflicting reports regarding whether this heterogenous pathogen is adapted to infecting particular host species. To what degree the variability described above contributes to host specialization or preference is still unclear. For some examples of generalist necrotrophic fungi such as *Botrytis cinerea*, there is evidence suggesting that selective pressure maintains moderate virulence against a broad spectrum of host species (Caseys et al., 2021). In a study by Koike et al. (2016), *M. phaseolina* isolates from melon, thyme, and apple were tested against strawberry and failed to cause disease. Moreover, five cover crop species tested against strawberry-derived isolates also did not develop symptoms. However, results from Su et al. (2001) indicate that no specialization occurs for isolates from sorghum, cotton, or soybean, and while corn isolates seemed to colonize corn roots better than other isolates, it also was the most virulent against soybean. Similar cross-host species pathogenicity experiments have been carried out testing hosts against isolates collected across species and geographic distances, but no isolate had restricted virulence to a single host (Mayék-Pérez et al., 2001; Reyes-Franco et al., 2006). Although there is some evidence to suggest that host preference exists for certain isolates, it is not certain whether this is the result of evolutionary specialization or an artifact of environmental factors, which may alter the virulence of the pathogen against specific hosts. Recently, a large-scale analysis of three *M. phaseolina* isolates from three different hosts revealed structural chromosomal rearrangements and SNPs, an indicator of genomic flexibility, although no pattern was necessarily associated with a particular host (Burkhardt et al., 2019). However, ten genes were identified to be exclusive to strawberry pathogenic isolates (Burkhardt et al., 2019). Whether these genes (alleles)? are the causal factor for the host preference of these isolates has not been reported.

### *M. phaseolina* -omics studies

In 2012, Islam et al. reported on the first draft *M. phaseolina* genome, from an isolate of infected jute. Cross-species homology analysis revealed its close synteny with another soil-borne generalist fungus, *Fusarium oxysporum*, which shares 54.10% of its predicted genes with *M. phaseolina*. Additionally, the authors identified 537 predicted proteins in the *M. phaseolina* genome that are included in the pathogen–host interaction database (PHI-base), a collection of experimentally verified virulence effectors from bacterial, fungal, and oomycete pathogens. So far, genomes of *M. phaseolina* isolates from a variety of hosts including jute, strawberry, alfalfa, sorghum, and castor have been sequenced (Islam et al., 2012; Verma et al., 2018; Burkhardt et al., 2019; Hossen et al., 2019; Purushotham et al., 2020; Marquez et al., 2021). Sequencing and assembly *M. phaseolina* genomes has led to comparative studies across isolates and geographic locations, and the development of new methods to study plant–*M. phaseolina* interactions to elucidate biological processes involved in pathogenicity.

Liu et al. (2022) used transcriptomic analysis to study the microsclerotia formation stages of *M. phaseolina*, revealing an enrichment of reactive oxygen species (ROS)-related genes among up-regulated differentially expressed genes.

The first documented proteomic analysis on *M. phaseolina* published by Zaman et al. (2020) compared the expression of proteins with and without challenge of the fungus by the jute endophytic bacterium *Burkholderia contaminans* NZ. A large number of hydrolyzing enzymes predicted in the *M. phaseolina* genome were confirmed to be expressed through this study. *M. phaseolina* co-cultured with this bacterial antagonist showed signs of decreased virulence, morphological changes, and altered metabolic processes switching to a more dormant state that prioritizes survival over growth. Characterization of the *M. phaseolina* mycelial proteome by Arafat et al. (2022) revealed a network of proteins that could be classified by their function including growth and reproduction, stress tolerance and virulence, nutrient synthesis, transcription and translation regulation, and more. Sinha et al. (2022) identified 117 proteins in the *M. phaseolina* secretome isolated from wheat bran samples, including cell wall degrading enzymes (CWDEs) and proteases which are vital for breaching plant defense mechanisms. Recently, Pineda-Fretez et al. (2023) identified 250 proteins secreted by *M. phaseolina* grown in liquid media supplemented with a soybean leaf infusion. Among these proteins were numerous CWDEs, and peptidases, as well as 54 putative effectors (Pineda-Fretez et al., 2023). These proteomic studies provide the groundwork for potential functional studies of specific genes or gene groups involved in virulence.

### Effects on host cell wall and membrane permeability

A notable characteristic of the *M. phaseolina* genome is the abundance of genes involved in cell wall degradation. Numerous carbohydrate-active enzymes (CAZymes) including glycoside hydrolases (GH), glycosyltransferases (GT), carbohydrate esterases (CE), carbohydrate-binding modules (CBM), and polysaccharide lyases (PL), were predicted within the genome. The *M. phaseolina* genome possesses high levels of GHs (four times more than GTs), and CEs (Islam et al., 2012), which is consistent with previous observations that this pathogen has greater cellulolytic activity than most other fungi (Kaur et al., 2012a). A β-1,4-endoglucanase produced by this pathogen was previously characterized and shown to be similar to plant-derived endoglucanases (Wang and Jones, 1995). While the significance of this is unclear, use of a CWDE that is similar to a plant-derived enzyme may allow for surreptitious modification of the host cell wall without eliciting plant defense responses. A repertoire of genes associated with lignin depolymerization, including laccases, lignin peroxidases, galactose oxidases, chloroperoxidase, haloperoxidases, and heme peroxidases were also identified. The *M. phaseolina* genome encodes the highest number of laccases in comparison to seven other fungal species (Islam et al., 2012). Production of these CWDEs has also been observed in *M. phaseolina* grown *in vitro* (Ahmad et al., 2006; Ramos et al., 2016). The activity of phosphatidases, which hydrolyze phosphatide lipids and alter the permeability of cellular membranes, was also observed during *Brassica juncea* infection with *M. phaseolina* isolates of various levels of virulence. A strong correlation between host susceptibility, pathogen virulence, and phosphatidase activity was described, which peaked at 18 days post-inoculation (Srivastava and Dhawan, 1982). The extensive collection of genes associated with cell wall and membrane disruption suggests that this pathogen is well equipped to breach diverse wall polysaccharide and lipid bilayer compositions, which differ between plant lineages (Kubicek et al., 2014). This perhaps explains, at least in part, *M. phaseolina*’s wide host range.

An unusually high number of amino acid transporters, which are likely useful for accessing host protein degradation products, were predicted in the *M. phaseolina* genome. In addition, enzymes involved in amino acid metabolism are enriched in the mycelial proteome (Arafat et al., 2022). Uptake of host-derived metabolites, such as amino acids, is likely to occur via hyphopodia of *M. phaseolina* formed during its biotrophic infection phase (**Figure 1A**) as well as from leaking host cells with degraded cell walls and plasma membranes during its necrotrophic phase.

### Signal transduction and host crosstalk

The *M. phaseolina* genome encodes a multitude of protein kinases and G-protein coupled receptors, some of which are known to facilitate host recognition in other pathogens (Islam et al., 2012). The abundance of such receptors and signal transduction components may contribute to the ability of *M. phaseolina* to recognize and utilize a great variety of plant species as hosts.

In fungi, redox signaling is essential for the regulation of developmental processes, crosstalk with plant hosts, and degradation of host lignocellulose (Breitenbach et al., 2015). *M. phaseolina* infection causes nitric oxide (NO) accumulation in jute (*Corchorus capsularis*), but the fungus itself is also capable of NO production *in vitro* (Sarkar et al., 2014). NO is a key signaling factor in the early stages of the host hypersensitive response (HR). In plants pathogen-induced NO production is accompanied by a burst of reactive oxygen species (ROS), such as superoxide (O_2_^−^) and hydrogen peroxide (H_2_O_2_) (Van Baarlen et al., 2004; Asai and Yoshioka, 2009). Balanced accumulation of both NO and H_2_O_2_ seems to be required to trigger HR-associated cell death (Delledonne et al., 1998; Delledonne et al., 2001), although there are somewhat conflicting reports as to whether NO and ROS act synergistically (Delledonne et al., 2001) or antagonistically (Asai and Yoshioka, 2009; Asai et al., 2010) in this process.

Interestingly, genes involved in ROS-related functions were found to be differentially expressed in *M. phaseolina* during microsclerotia formation, suggesting that ROS, specifically O_2_−, stimulates microsclerotia formation (Liu et al., 2022). Moreover, studies across multiple filamentous fungi have shown that ROS have functions in cell differentiation and development in fungi (Breitenbach et al., 2015). *In vitro* treatment of cultures with ROS further supported this hypothesis. A fascinating possibility is that ROS produced by *M. phaseolina* may interfere with ROS/NO-controlled processes in its hosts and *vice versa*. Pathogen-derived ROS and NO may contribute to HR in the host, which may increase its virulence during necrotrophy, while the defense-linked oxidative burst in the host may contribute to *M. phaseolina* microsclerotia formation.

### Secondary metabolites

Fungi produce diverse bioactive natural products, some of which enhance their virulence, such as mycotoxins (Chooi and Tang, 2012). A total of 75 putative secondary metabolite-related genes were identified in the *M. phaseolina* genome, which is more than double of what can be found in each of the plant pathogenic fungi *Magnaporthe grisea*, *B. cinerea*, *Sclerotinia sclerotiorum*, and *Fusarium graminearum*. These include genes encoding polyketide synthases (PKS), non-ribosomal peptide synthases (NPRS) and PKS-NPRS hybrids, which synthesize precursors to various secondary metabolites including those involved in virulence (Islam et al., 2012). Additional expression analyses and functional studies are required to confirm the role of these genes. Examples of secondary metabolites produced by *M. phaseolina* include phaseolinone (Siddiqui et al., 1979; Dhar et al., 1982), phaseolinic acid (Mahato et al., 1987), (−)-botryodiplodin (Ramezani et al., 2007; Abbas et al., 2019), succinic acid, tyrosol, (R)-mellein, *cis*-(3R,4R)-4-hydroxymellein, azelaic acid (Salvatore et al., 2020), kojic acid, moniliformin, orsellinic acid (Khambhati et al., 2020), macrollins A–C (Gao et al., 2021), phaseocyclopentenones A and B, and guignardone A (Masi et al., 2021). However production of such metabolites seems to be dependent on a number of factors including isolate variability and the presence of host substrates. To what extent virulence is affected by these metabolites is largely unknown.

Phaseolinone was described to be a novel phytotoxin when it was first identified in *M. phaseolina* and has been documented to affect plant seed germination, wilting, and leaf necrosis (Dhar et al., 1982; Bhattacharya et al., 1992; Bhattacharya et al., 1994). The compound (−)-botryodiplodin is a well-documented toxic metabolite prevalent in ascomycetes such as *M. phaseolina*. When soybean plants were infected with *M. phaseolina*, this compound was isolated exclusively from root tissues displaying charcoal rot symptoms (Ramezani et al., 2007; Abbas et al., 2019), suggesting that it is produced during pathogenesis. In these assays, however, phaseolinone could not be detected in any soybean tissue examined despite previous reports describing it as a metabolite unique to *M. phaseolina*. Analysis by Shier et al. (2007) provides several possible reasons for this discrepancy, one being that Dhar et al. mistakenly characterized (−)-botryodiplodin as phaseolinone. Another possible explanation is that different isolates of *M. phaseolina* produce different “cocktails” of toxins. This is supported by the fact that mellein was identified in some, but not all, isolates of this pathogen recovered from symptomatic soybean plants collected in Mississippi, U.S. (Khambhati et al., 2020). The abundance of phytotoxic secondary metabolites potentially contributing to *M. phaseolina* pathogenicity is mirrored by the induced expression of numerous detoxification-related genes in *A. thaliana* after infection with this pathogen (Schroeder et al., 2019).

## Host immunity and defense mechanisms

Plants have innate defense mechanisms that are widely conserved across species, and which allow for protection against a wide range of pathogens. The fact that within given host species varieties or cultivars with different levels of resistance against *M. phaseolina* have been described, strongly suggests the existence of plant immune mechanisms against this pathogen. Multiple efforts have been made to uncover the genetic basis of *M. phaseolina* resistance in crop species. Despite these recent advancements, molecular mechanisms that determine host resistance against *M. phaseolina* are still poorly understood.

One of the first lines of defense that plants possess is a physical barrier that simultaneously functions as a sensory organ – the cell wall. It can efficiently deter many pathogens, contributing to non-host resistance, an unspecific type of immunity protecting the plant against a wide-range of pathogens. Polysaccharides of diverse compositions form a network that supports the structural integrity of the cell wall, alongside proteins, which have roles in the remodeling and turnover of cell wall components. For pathogens that can breach and decompose this barrier, the cell wall may be a valuable nutrient source. However, disruptions in the integrity of the cell wall, as well as the activity of enzymes that cause its degradation, including those that originate from the host itself, are known to trigger signaling cascades eliciting biotic stress responses. Evidence for the role of cell wall maintenance in host defense is thoroughly reviewed by (Hamann, 2012). Molecular signals released as a result of cell wall damage can be perceived as damage-associated molecular patterns (DAMPs) by pattern recognition receptors (PRRs) residing on the plant plasma membrane (**Figure 1A**) leading to a robust local defense response called pattern triggered immunity (PTI) and possible systemic defense signaling (Hou et al., 2019).

PRRs can also detect conserved pathogen-associated molecular patterns (PAMPs) directly derived from invading pathogens and activate PTI (**Figure 1A**) (Bigeard et al., 2015). For example, the *A. thaliana* PRR FLS2, a receptor-like protein kinase (RLK), responds to flagellin, a ubiquitous bacterial PAMP conferring PTI (Gómez-Gómez and Boller, 2000). Responses to PAMP recognition include ionic influxes in the cytosol, increased ROS and NO production, activation of mitogen-activated protein kinase cascades, and transcriptional reprogramming that entails induction of pathogenesis related (*PR*) genes (Nürnberger et al., 2004). In turn, some pathogens have adapted to counter host defenses through proteins and secondary metabolites collectively called effectors that can interfere with PTI. Effectors can serve as toxic agents themselves or as a means to evade and suppress host defense mechanisms (Lo Presti et al., 2015). Although there are several characteristics common to many effector proteins, such as size, species exclusivity, and expression profile, exceptions are also common. In terms of total secreted proteins, hemibiotrophs on average have the greatest number of effectors than any other fungal lifestyle group (Lo Presti et al., 2015).

The perpetual coevolution of plant hosts and microbial pathogens, collectively described as the “zigzag model”, has contributed greatly to the repertoire of attack/defense mechanisms encoded by the genomes of both sides involved (Chisholm et al., 2006; Jones and Dangl, 2006). Pathogen effectors can be directly or indirectly identified by specific receptors encoded by host resistance (*R*) genes which then trigger an acute immune response that often results in the “hypersensitive response” (HR) a defense mechanism involving localized program cell death of plant cells. Many fungal avirulence (*Avr*) genes encoding Avr proteins have gene-for-gene relationships with plant host *R* genes and typically act as effectors. This tertiary layer of defense is called effector-triggered immunity (ETI) (Macho and Zipfel, 2014; Lo Presti et al., 2015). The *A. thaliana* ecotype Col-0 possesses approximately 165 NB-LRR genes, which is the largest class of R genes characterized by their nucleotide binding (NB) and leucine rich repeat (LRR) domains (Dangl and Jones, 2001; Wei et al., 2020). PTI and ETI make up the plant’s inducible defense core.

### PAMP candidates of *M. phaseolina*

Eight orthologs of the *Phytophthora parasitica* cellulose-binding elicitor lectin (CBEL) gene, which has a role in the perception of host cell wall components and is a known PAMP, were found in the *M. phaseolina* genome (Gaulin et al., 2002; Islam et al., 2012). The 13 amino acid comprising peptide Pep-13 is a known PAMP motif in cell wall transglutaminases from *Phytophthora sojae* (Brunner et al., 2002). Three homologs containing the Pep-13 motif are found in *M. phaseolina* (Islam et al., 2012). As several *M. phaseolina-*derived PAMPs have been identified thus far, it is likely that plants recognize the presence of this pathogen through mechanisms similar to those of other pathosystems.

#### Phytohormones

Three phytohormones are crucial for the orchestration of plant defense responses: salicylic acid (SA), jasmonic acid (JA), and ethylene (ET) (Glazebrook, 2005; Bari and Jones, 2009). SA accumulates in host tissues, can trigger HR and comprehensive transcriptional reprogramming as a response to infection, and mediate efficient defense against biotrophic and hemibiotrophic pathogens. It is also known to induce systemic acquired resistance (SAR), in which distal tissues acquire heightened resistance to secondary infection due to elevated expression of pathogenesis related (*PR*) genes (Ross, 1961; Durrant and Dong, 2004). JA and ET are associated with comprehensive defense responses against necrotrophic pathogens (Bari and Jones, 2009). Similar to, but independent from SA, the phytohormones JA and ET are also known to trigger a systemic defense response called induced systemic resistance (ISR) (Pieterse et al., 1996). Although signaling pathways controlled by these plant hormones are tuned to defend against pathogens of distinct lifestyles, they are known to affect each other both synergistically and antagonistically (Clarke et al., 2000; Thomma et al., 2001; Mur et al., 2006; Koornneef and Pieterse, 2008). Cross-talk between phytohormone-signaling pathways allows the host plant to balance the energy cost of pathogen defense to that of its own growth and propagation (Pieterse et al., 2012).

Transcriptomic analysis in *A. thaliana* roots revealed that *M. phaseolina* induces JA-, ET-, and SA-responsive genes, which is consistent with the observation that this pathogen leads a hemibiotrophic lifestyle. Mutants compromised in signaling mediated by each of these three hormones exhibited increased susceptibility to *M. phaseolina* (Schroeder et al., 2019). *M. phaseolina* induced upregulation of JA- and ET-responsive genes has been reported in several species of hosts susceptible to this pathogen, both in the shoot and the root (Gaige et al., 2010). In *Sesamum indicum*, widely known as sesame, SA responsive differential gene expression in the root was in correlation with the early biotrophic phase of *M. phaseolina* infection both in resistant and susceptible varieties, although overall transcript levels were greater in the resistant variety (Chowdhury et al., 2017). Accumulation of JA- and ET-signaling gene transcripts was seen during the BNS and through the necrotrophic phase in these studies (**Figure 1C**). Moreover, the authors conducted a priming assay with each of these phytohormones and found that pre-treatment of sesame with JA and ET significantly reduced *M. phaseolina*-associated disease symptoms, while JA- and ET-inhibitors significantly increased susceptibility to this pathogen. Taken together, the evidence suggests that the timing and amplitude of SA, JA and ET signaling are critical in determining the susceptibility of the host. While all three of these defense-associated phytohormones seem to cooperate in protecting host roots against *M. phaseolina*, it is important to consider that results based on gene expression studies in whole organs reflect responses that are averaged across different cell types. Cell-type specific regulation of phytohormone-mediated defense has not been explored in *M. phaseolina* pathosystems.

#### ROS

As introduced earlier, plants produce ROS, primarily superoxide (O_2_−) and hydrogen peroxide (H_2_O_2_), in response to pathogen invasion. H_2_O_2_ resulting from such an oxidative burst can act as a diffusible signal in plants that activates defense-related genes and other defense reactions, ultimately triggering local HRs (Levine et al., 1994). The level of ROS in plant tissue is enzymatically controlled by superoxide dismutases (SOD), which catalyze the conversion of O_2_^−^ to H_2_O_2_ and oxygen (Wang et al., 2018). When interactions of sesame plants with *M. phaseolina* were studied, the susceptible cultivar VRI-1 showed consistently higher accumulation of H_2_O_2_ in comparison to the resistant Nirmala variety, following the BNS (Chowdhury et al., 2017). Consistently, less lipid peroxidation and higher accumulation of *SiSOD* was observed during the necrotrophic phase in the resistant variety (Chowdhury et al., 2017).

Asai and Yoshioka (2009) found that necrotic lesions caused by *Botrytis cinerea* on *Nicotiana benthamiana* were reduced when the oxidative burst was suppressed, while disease lesions expanded when NO production was inhibited. HR induction in the host plant is favorable for pathogens during their necrotrophic phase, when they feed on dead tissue, especially when the HR is uncontrolled, as is expected if the pathogen produces additional ROS during infection (Sarkar et al., 2014; Liu et al., 2022). It has also been shown that changes in NO levels caused by *M. phaseolina* infection in sesame were dependent on the susceptibility of the cultivar, with resistant varieties exhibiting a rapid but moderate increase in NO, while susceptible varieties showed a delayed and robust decrease in NO (Acharya and Acharya, 2007). Similarly, in interactions of the necrotrophic fungus *Sclerotinia sclerotiorum* with *A. thaliana*, rapid and transient accumulation of NO was observed in resistant ecotypes, while NO accumulation was also observed in susceptible ecotypes at later time points (Perchepied et al., 2010). *M. phaseolina*-triggered accumulation of H_2_O_2_, however, was found between 24 and 72 hpi to be consistently higher in susceptible sesame genotypes compared to resistant ones (Chowdhury et al., 2017). At later time points when the pathogen has presumably entered its necrotrophic phase, resistant genotypes showed signs of ROS detoxification. Taken together these observations suggest that the timing and amplitude of NO and ROS production, interactions of these molecules and their turn-over are likely factors contributing to host susceptibility during necrotrophic phases of pathogen infections. While the oxidative burst is known to contribute to PTI and ETI against biotrophs, the degree of host resistance seems counter-correlated with ROS levels during necrotrophic infection phases.

#### Secondary metabolites

Like *M. phaseolina*, plants produce a diverse array of secondary metabolites with roles in biotic interactions. Antimicrobial secondary plant metabolites synthesized in response to biotic stress are collectively called phytoalexins (Ahuja et al., 2012), while antimicrobial compounds constitutively present in plants are referred to as phytoanticipins (Tiku, 2020). Potential phytoalexins and phytoanticipins found in *A. thaliana* include phenylpropanoids, glucosinolates, terpenoids, and camalexin (Kliebenstein, 2004). Consistent with the likely roles of host secondary metabolites as toxins against *M. phaseolina*, the genome of this fungus contains a large set of detoxification genes, including genes encoding dehydrogenases, acyl-coA N-acetyltransferases, monooxygenases, and cytochrome P450s (Islam et al., 2012).

One of the major plant metabolic pathways often discussed in relation to pathogen defense is the phenylpropanoid pathway, which is required for the synthesis of diverse phenolic metabolites including lignols, flavonoids, isoflavonoids, and tannins (Fraser and Chapple, 2011; Rehman et al., 2012). Soybean produces a repertoire of phytoalexins, including the isoflavonoid derivatives glyceollins, during both symbiotic and pathogenic microbial entry. Work by Lygin et al. (2013) showed that the downregulation of isoflavonoid biosynthesis, and consequently glyceollins, significantly increases charcoal rot disease severity in soybean. In *Medicago truncatula*, genes involved in the phenylpropanoid pathway, as well as flavonoid and isoflavonoid biosynthesis were up-regulated dramatically in the shoot but not in the root in response to *M. phaseolina* infection at 24 and 48 hpi (Gaige et al., 2010). As the authors pointed out, lack of induction of these genes in roots could be the result of pathogen suppression or tissue-specific gene regulation. Nonetheless this shows that *M. phaseolina*, despite being a soil-borne pathogen, strongly induces systemic upregulation of secondary metabolite synthesis genes. Accumulation of phenolic compounds synchronized with the induced activity of phenylalanine ammonia lyase (PAL), a key player phenylpropanoid pathway, was also seen in the root tissue of plate-grown sesame after infection (Chowdhury et al., 2017). Notably, this induction was stronger in the resistant Nirmala variety and it only occurred after the BNS. It is unknown according to this paper whether similar induction occurred in the shoot tissue. Radadiya et al. (2021) also observed upregulation of genes encoding key phenylpropanoid pathway enzymes in sesame roots post inoculation with *M. phaseolina* in soil through transcriptome analysis. Both Chowdhury et al. (2017) and Radadiya et al. (2021) reported earlier upregulation of these genes in different resistant varieties, and a delayed response in the susceptible varieties, suggesting that there is a correlation between timing of defense responses and susceptibility to this pathogen.

Glucosinolates, another major class of secondary metabolites, are known for their abundance in plants of the Brassicaceae family, which includes the model plant *A. thaliana* and several economically important crops like oilseed rape (*Brassica napa*). The glucosinolate-myrosinase system is a defense mechanism that releases toxic glucosinolate derivatives upon activation by physical damage to plant tissue (Wittstock and Halkier, 2002). This is commonly perceived as a primary defense against herbivores, but it is also known that some isothiocyanates, which are products of the glucosinolate-myrosinase reaction, have fungitoxic properties (Drobnica et al., 1967; Tierens et al., 2001; Smolinska et al., 2003). Cruciferous soil amendments have been used to successfully suppress generalist hemibiotrophic pathogens like *F. oxysporum* growth and protect crops from disease (Rosa and Rodrigues, 1999), however, it is still unclear whether this effect is the product of chemically active inputs or that of overall changes in the soil microbiome. Brassica seed meal amendment trials with strawberry have had limited success in protecting plants from *M. phaseolina* (Agostini, 2011; Mazzola et al., 2017). It remains to be demonstrated whether glucosinolate activity in known brassicaceae hosts like *A. thaliana*, *Brassica napa*, and *Brassica juncea* play a role in resistance against *M. phaseolina*.

#### Root-specific defense

*M. phaseolina* is a soil-borne pathogen, which means that the most accessible point of entry for hyphae will be in the root of host plants. While it is widely accepted that the two major plant defense hormones SA and JA act largely mutuality antagonistic, with each pathway corresponding to a particular pathogen life cycle (Glazebrook, 2005), several studies specifically focusing on root responses paint a more complex picture. Inoculation of *Phytophthora parasitica* on *A. thaliana* caused a simultaneous upregulation of both SA and JA pathway genes in the root (Attard et al., 2010). *Fusarium oxysporum* infection repressed many known defense-associated genes in roots, and only three genes overlapped with previously reported microarrays with inoculated leaves (Chen et al., 2014).

It is therefore necessary to mention that generalizations on plant immune responses should be made with caution if the data is derived from whole plants or even whole organs. For example, when JA is applied to *Brassica oleracea* to mimic wounding, the expression and regulation of known defense mechanisms like secondary metabolite synthesis and phytohormone responses varied depending on tissue type examined and the site of treatment (Tytgat et al., 2013). Root-specific pathogen-induced responses have been reviewed thoroughly by Chuberre et al. (2018), but it has yet to be confirmed whether defense responses to *M. phaseolina* are also tissue specific. In sorghum, *M. phaseolina* triggered a greater induction of chitinase genes in the aerial tissue in comparison to roots (Sharma et al., 2014).

## Research on *M. phaseolina*–host relationships and future perspectives

Despite the long history of growers losing crop yield to disease caused by *M. phaseolina*, sustainable and effective protection strategies against this pathogen are still lacking. Several factors contribute to the obstacles prohibiting the advancement of research in this field, including the high diversity of this pathogen, and the susceptibility of field-generated data to environmental variability. Recently, a high-throughput pathosystem using the model plant *A. thaliana* has been established, which will likely accelerate research in this field (Schroeder et al., 2019). In addressing pathogen variability, it seems unusual that a monotypic genus can represent the level of genetic and morphological diversity seen in *M. phaseolina* without evidence of sexual reproduction. *F. oxysporum* also has not been documented to undergo sexual reproduction but is suspected to preserve its diversity through horizontal gene transfer (Gordon, 2017). Research has led to the following hypothetical scenario: virulent genotypes emerged through significant selection pressures such as agricultural activity while recent relatives that are capable of outcrossing, possibly within populations existing today, sustain a “reservoir” gene pool (Gordon, 2017). Although there is no direct evidence that *M. phaseolina* fit this model, this analysis may provide critical insights to the evolution of *M. phaseolina*, considering its striking similarity to *F. oxysporum* (Islam et al., 2012).

There are decades of records documenting *M. phaseolina* pathogenicity on a wide range of crop species, including soybean (Smith and Carvil, 1997; Coser et al., 2017; da Silva et al., 2019a), sesame (Chowdhury et al., 2017; Bedawy and Moharm, 2019; Radadiya et al., 2021; Yan et al., 2021), strawberries (Koike, 2008; Burkhardt et al., 2019; Nelson et al., 2021), and more. The advancement of high-throughput sequencing and genome-wide analysis technologies have allowed for the identification of many resistance-associated loci in cultivars of various host species, sampled from infected fields across the globe. Previously, Muchero et al. (2011) identified 9 QTL regions tied to *M. phaseolina* resistance in cowpea (*Vigna unguiculata*) through QTL mapping with a recombinant inbred line population. These regions include markers and genes associated with disease resistance homologous to genes in other species including soybean and medicago. Within the candidate gene sets identified, there is a noticeable presence of genes involved in pectin metabolism. A bimodal distribution of disease incidence was observed in castor (*Ricinus communis*), which suggests that a genetic variant with major effects on the disease resistance trait is present in the population (Tomar et al., 2017). The authors discovered three QTLs through linkage mapping with the most significant region predicted to explain 71.2% of the phenotypic variation, which combined with the phenotypic distribution supports the existence of major effect genes.

More recently, genome-wide association has been used to identify resistance loci in species with available reference genomes. Coser et al. (2017) conducted a disease screen using 459 soybean lines to identify *M. phaseolina* resistance loci through genome-wide association. In field and greenhouse experiments, a total of 19 SNPs were identified to have a significant association with the resistant phenotype. Orthologous gene regions to *A. thaliana* were also identified, and these included genes involved in biotic and abiotic stress response, cell wall composition, and ethylene signaling. Surprisingly, there were no overlaps between these SNP regions and QTL regions identified by da Silva et al. (2019b) using QTL-seq, which combines bulk segregant analysis with next-generation sequencing to statistically link loci to a quantitative trait. This could be because the segregating population used for QTL-seq was derived from parental lines (PI567562A and PI567437) that were not included in the set tested by Coser et al. Nonetheless, it should be taken into consideration that factors such as treatment conditions, sequencing methods, and genotypes of both host and pathogen could yield variable QTL. Nelson et al. (2021) identified three loci linked to *M. phaseolina* resistance from a breeding population obtained from controlled crosses of commercial strawberry. Many gene regions that exhibit strong associations with disease phenotype have not been narrowed down to candidate genes nor have they been assessed for their function. Consequently, evidence for a strong causal relationship between specific genes or molecular mechanisms and *M. phaseolina* resistance is still lacking. Cross-species synteny analysis may be a useful tool in identifying conserved trait loci.

Experimentation with *M. phaseolina* has primarily been focused on the field. However recently greenhouse studies have allowed for observation of disease progression in more controlled environments. Young sesame seedlings were inoculated in mycelium mixed soil and kept in artificial climate boxes in a study by Yan et al. (2021) and root tissue was collected for RNA-seq. Schroeder et al. (2019) used agar plates for assessing *M. phaseolina* interaction with small hosts like *A. thaliana* seedlings. While many studies have used pre-infected plots to study the relationship between native *M. phaseolina* and host crops (Miklas et al., 1998; Tomar et al., 2017), various methods of pathogen inoculation have been employed, including mixing mycelium in soil (Bedawy & Moharm, n.d.; Mahmoud et al., 2018), dipping roots in mycelium (Nelson et al., 2021), cut-stem inoculation (Lygin et al., 2013; Pawlowski et al., 2015), and incubating seeds with mycelium (Coser et al., 2017). It has not been evaluated whether the method of pathogen introduction to the host has an effect on the outcome of the infection. Commonly used metrics of disease susceptibility include necrotic lesion size, percent mortality, or overall disease symptom severity quantified by a numeric scoring scale. In order to evaluate the most suitable screening method for *M. phaseolina* resistance, Mengistu et al. (2007) tested five different disease metrics in soybean for their consistency across experimental years and environments. Colony-forming unit index (CFUI), calculated by counting colony forming units (CFU) derived from a set amount of ground tissue and dividing by the CFU count of the most susceptible genotype (highest CFU) within the experiment, was deemed to be the most consistent across all experimental conditions. This was followed by root and stem symptom severity (RSS), determined by discoloration of stem and taproot tissue, which the authors noted was a less time and resource consuming alternative disease assessment method (Mengistu et al., 2007).

## Conclusions

Movement towards reduced chemical use in agriculture is an imperative step towards sustainability. In order to develop resistant varieties of crops, it is necessary to first elucidate functions of specific genes/gene groups and quantify their contribution to host resistance and pathogen virulence. With modern sequencing technologies, high-throughput assay capabilities, and decades of research on *M. phaseolina* pathology, researchers are equipped to advance our understanding of generalist hemibiotrophic pathogens like *F. oxysporum* and *M. phaseolina*, which are notoriously difficult to control. Slowly, we are beginning to understand why *M. phaseolina* is a successful pathogen. First, its wide host range and prevalence throughout the globe indicate high genomic plasticity that has allowed this fungus to adapt to local conditions and host plant species. This is supported by the presence of repetitive sequences and transposable elements within its genome. Moreover, no evidence for sexual reproduction has been documented for *M. phaseolina* thus far. This is striking considering the level of genomic variability seen across isolates even from the same host species and geographic origins, and evidence for host specificity seen in multiple examples. Several possible explanations including horizontal gene transfer, sporadic mutations mediated by TEs and repeats, and epigenetics could be hypothesized, but nonetheless it is likely that the instability of the *M. phaseolina* genome contributes to its extensive virulence capabilities. It is also important to note that the sampling of *M. phaseolina* in published studies is overwhelmingly biased towards those collected from agricultural fields and may not be an accurate representation of naturally occurring populations. Recently, more and more studies describing *M. phaseolina* pathosystems, using various isolates and host species, from various levels of genetic regulation are becoming available.

Unfortunately, it seems that the likelihood of finding crop genotypes conferring complete immunity to this pathogen is very low. To date, no specific *R*-*Avr* gene interactions have been identified in any host interactions with *M. phaseolina*. Resistance, if seen at all, seems to be a quantitative trait. Several recurring themes can be identified in the literature, such as: cell wall metabolism, oxidative stress and detoxification. These known plant immune responses potentially contribute partially to the overall disease phenotype. Through what mechanisms, and to what extent these biological processes impact the phenotype remains to be answered. As *M. phaseolina* is a generalist pathogen, it may benefit researchers to view defense against this pathogen from a generalist perspective.

Assuming that host species of this pathogen possess similar basic forms of defense and that canonical plant defense-related signal transduction pathways are intact, temporal parameters are likely to determine the fate of the host plant: the speed at which host reacts to the pathogen, and the duration of the subsequent response. Moreover, it must be taken into account that each cell in the plant experiences pathogen invasion differently and that systemic defense activation is a gradient. Neither of these temporal parameters, to our knowledge, have been studied at the tissue nor cell type-specific level in *M. phaseolina* pathosystems. Spatio-temporal analysis of host defense gene activation, as well as an in-depth examination of the *M. phaseolina* secretome, are potential avenues of further research in this field.

## Author contributions

TE: Conceptualization, Funding acquisition, Supervision, Writing – review & editing. MS: Conceptualization, Writing – original draft, Writing – review & editing.
